# Pyroptosis is related to immune infiltration and predictive for survival of colon adenocarcinoma patients

**DOI:** 10.1038/s41598-022-13212-2

**Published:** 2022-06-02

**Authors:** Huiwen Lu, Yimeng Sun, Zirui Zhu, Junqiao Yao, Huimian Xu, Rui Huang, Baojun Huang

**Affiliations:** 1 Department of Surgical Oncology and General Surgery, The First Hospital of China Medical University, Key Laboratory of Precision Diagnosis and Treatment of Gastrointestinal Tumors (China Medical University), Ministry of Education, No.155 Nanjing North Street, Heping District, Shenyang, 110001 People’s Republic of China; 2 Department of Clinical Medicine of Year 2017, Dalian Medical University, Dalian, People’s Republic of China

**Keywords:** Tumour biomarkers, Cancer screening

## Abstract

Pyroptosis is a novel type of programmed cell death, initiated by inflammasome. Pyroptosis inhibits the development and metastasis of colon cancer and is associated with patients’ prognosis. However, how the pyroptosis-related genes predict the survival of patients is still unclear. In the study, colon adenocarcinoma (COAD) patients were divided into two groups according to the expression of pyroptosis-related regulators through consensus clustering. DEGs between two clusters were analyzed by using COX and Lasso regression. Then, regression coefficients in Lasso were used to calculate the risk score for every patient. Patients were classified into two types: low- and high-risk group according to their risk score. The difference of immune microenvironment infiltration and clinicopathological characteristics between subgroups was performed. Moreover, the nomogram model was built on the bases of risk model and clinicopathological factors. The TCGA-COAD cohort and GEO cohort were used as training and validating set respectively. 398 COAD patients in TCGA training set were identified as two regulation patterns via unsupervised clustering method. Patients in cluster 2 showed better prognosis (*P* = 0.002). Through differentiated expression analysis, COX and Lasso regression, a 5-gene prognostic risk model was constructed. This risk model was significantly associated with OS (HR: 2.088, 95% CI: 1.183–3.688, *P* = 0.011), validated in GEO set (HR:1.344, 95%CI: 1.061–1.704, *P* = 0.014), and patients with low risk had better prognosis (*P* < 0.001 in TCGA; *P* = 0.038 in GEO). Through ROC analysis, it can be found that this model presented better predictive accuracy for long-term survival. Clinical analyses demonstrated that high-risk group had more advanced N stage, higher risk of metastasis and later pathological stage. Immune-related analysis illustrated that low-risk group had more immune cell infiltration and more activated immune pathways. The pyroptosis-related risk model can be predictive for the survival of COAD patients. That patients with higher risk had poorer prognosis was associated with more advanced tumor stage and higher risk of metastasis, and resulted from highly activated pro-tumor pathways and inhibited immune system and poorer integrity of intestinal epithelial. This study proved the relationship between pyroptosis and immune, which offered basis for future studies.

## Introduction

Colon adenocarcinoma is one of the leading causes of cancer-related death^1^. Because most COAD patients were diagnosed at an advanced stage, their prognosis is poor^2^. Achievement of early diagnosis and individual treatment are considered as the most effective way to improve the survival of cancer patients. In clinical practice, some pathological factors, such as TNM stage and tumor differentiation, are widely used to predict the prognosis of colon adenocarcinoma patients^3^. However, the accuracy of clinical estimation is relatively low and precise treatment is limited because pathological factors fail to provide complete prognostic information. Thus, more tumor indicators or risk models predictive for intrinsic biology of the tumor in addition to pathological factors are necessary to evaluate tumor progression more accurately and promote the early diagnosis.

Pyroptosis, a type of inflammatory programmed cell death (PCD), mostly initially activated by microbial infections, is different from apoptosis, which as the most well-known way of PCD has been widely studied in past years^4^. Pyroptosis is promoted by inflammasome, a larger protein complex which is composed of a NOD-like receptor, caspase-1, and the adaptor protein ASC^5^, resulting in rapid pore formation on the cell membrane by its cleavage of Gasdermin-D (GSDMD), as well as inducing inflammatory reaction by converting the pro-forms of inflammatory cytokines IL-18 and IL-1β to their mature forms and further recruiting multiple inflammatory cells^6–9^. Caspase 4,5,11 can be activated directly by bacteria, leading to cell pyroptosis, called non-classical pathway of pyroptosis^9^.

Considering that inflammasomes play an important role in keeping the integrity of intestinal epithelium and promoting immune surveillance, dysregulation of pyroptosis is associated with some kinds of bacteria-triggered intestinal inflammation and occurrence of tumors^10,11^. Pyroptosis can inhibit the proliferation and metastasis of some types of cancer, mainly including gastric, colon, breast, ovarian and liver cancers^12^. Inflammasome-associated proteins, such as NLRs and ALRs, respond rapidly to various signals, especially microbes invading the gut, highlighting their importance for diseases, including colitis and colorectal cancer^11^. IL-18 and IL-1β, activated by pyroptosis-related pathways responsible for inducing inflammation and keeping the integrity of epithelial barrier, showed their protective role against colitis-associated colon adenocarcinoma^4,13,14^.

It can be concluded that pyroptosis plays a key role in carcinogenesis and tumor metastasis, especially for colon tumor^12,15^. However, whether pyroptosis-related genes or regulators can be predictive for the prognosis of colon adenocarcinoma patients is inconclusive. In this article, we aim to build a pyroptosis-related-regulator model to predict the prognosis of colon adenocarcinoma patients and provide the potential therapeutic target. To achieve it, 398 colon adenocarcinoma patients were clustered according to the expression of pyroptosis-related genes which were downloaded from The Cancer Genome Atlas (TCGA) database and two kinds of subtypes associated with prognosis were identified. Univariable and multivariable COX regression as well as the least absolute shrinkage and selection operation (LASSO) used to screen the prognosis-related regulators and further build an optimal risk model, which was validated in Gene Expression Omnibus (GEO) cohort. This risk model can predict prognosis and immune infiltration accurately. Then, gene set enrichment analysis (GSEA) was used to show the difference of key signaling pathway between high and low risk group. Finally, nomogram which combined with clinical pathological factors and prognostic genes was established to comprehensively predict patients’ survival. Taken together, this study showed the predictive value of pyroptosis-related regulators and provided potential prognostic biomarker for colon adenocarcinoma.

## Materials and methods

### Data collection

All data related to human subject were collected from public datasets, including TCGA and GEO. Colon adenocarcinoma RNA-seq data and clinical information were downloaded from TCGA database (https://portal.gdc.cancer.gov/). Validating data sets, GSE17526 and GSE39582 were obtained from GEO database (https://www.ncbi.nlm.nih.gov/geo/). Pyroptosis-related genes were collected from published articles, and were listed in supplementary Table S1.

### Data processing

The training set only included the TCGA colon adenocarcinoma (COAD) samples with complete clinicopathological data and prognostic data. Regarding the GEO data, raw data was processed with RMA background correction using affyPLM package to transform them as gene-expressed set which can be calculated, and then merged into one set. Next, limma package was used to reduce the batch effect on the merged dataset. Thus, validating data from two GEO microarrays could be analyzed synchronously. Additionally, another batch normalization was performed between TCGA training set and GEO validating set before construction and validation of risk model, so that they were comparable.

### Screening for differentially expressed genes (DEG)

Genes obtained from three data sets (TCGA, GEO, pyroptosis-related gene set) were intersected to identify the genes which were presented simultaneously in these three gene sets and further screen for the differentially expressed genes. The differentially expressed DEGs were identified within TCGA-CC data set by using limma package in R software (version 4.0.4) (adjusted *P* < 0.05). Volcano plots and heat maps were created by ggplot and pheatmap packages in R. Gene Ontology (GO) and Kyoto Encyclopedia of Genes and Genomes (KEGG) were performed to explore the enrichment in biological function and signaling pathway of these DEGs^16–18^.

### Applying consensus clustering

Consensus clustering was applied to construct pyroptosis-related patterns based on the expression of pyroptosis regulators using k-means methods. Clustering algorithm obtained from “ConsensuClusterPlus” package was used to determine the numbers and stabilities of clusters, and 1000 times repetitions were performed to verify the stability of our clusters. Kaplan–Meier curve and log-rank test analysis were used to analyze the survival difference between clusters, and the principal component analysis (PCA) was applied to verify its validity.

### Construction of pyroptosis-related regulators signature using LASSO regression

The DEGs were identified between two clusters (|logFC|> 1.0, adjusted *P* < 0.05) and further were analyzed by univariable COX regression to identify the association between DEGs and OS (*P* value < 0.05). Later, lasso regression analysis was performed to build an optimal prognostic model because of its function on decreasing the false positives in variables. Only genes with non-zero coefficients in lasso regression were used to calculate the risk score.

### Construction of a risk score model and gene set enrichment analysis (GSEA)

The prognosis-related DEGs were selected based on the result of lasso regression analysis. Risk score of each patient in TCGA cohort was calculated as follows:

$${\text{Risk score}} = \mathop \sum \limits_{i = 1}^{n} \left( {coefi*expri} \right)$$

The variables coef_i_ and expr_i_ respectively represent the lasso regression coefficients of gene i and the relative expression of the genes for patient i. Patients were divided into high- and low- risk group according to the risk score (the median risk score was regarded as cut-off point). Kaplan–Meier curve and log-rank test were used to analyze the survival difference between two groups respectively in training and validating data sets. A *P*-value < 0.05 was referred as significant. Meanwhile, GSEA was performed to identify the different signaling pathway between high and low risk groups and plot was showed. Additionally, ssGSEA was constructed by “GSVA” package to calculate the scores of infiltrating immune cells and estimate the immune-related functions. The ESTIMATE algorithm was adopted to determine the TME scores for patients.

### Validating the pyroptosis-related COAD gene signature

The patients in training and validating groups have been respectively divided into high and low risk groups based on risk score using the same cut-off point. Then, time-dependent receiver operating characteristic (ROC) curve analysis was performed by survival ROC package in R for calculating 1-year, 3-year and 5-year overall survival to validate the accuracy for survival prediction of our model in TCGA training datasets.

### Association between the pyroptosis-related gene signature and clinicopathological characteristics

Patients’ clinicopathological characteristics were downloaded from TCGA and GEO datasets, including age, gender, tumor location, pathological TNM stage. Continuous variables between high- and low-risk group were compared using the t test or one-way ANOVA, and Fisher’s exact test or the chi-square test was used to compare the differences between categorical variables. Univariable and multivariable Cox proportional hazards regression models were used to determine independent prognostic factors for colon adenocarcinoma patients, and the hazard ratio (HR) and its 95% CI were estimated. Aforementioned statistical analyses were performed using SPSS 22.0 statistical software. The *P*-value less than 0.05 was considered to be statistically significant.

### Development and validation of a predictive nomogram

Nomogram model was built based on the risk score and clinicopathological features so as to predict the prognosis of COAD patients more accurately. Variants included in nomogram were determined by univariable and multivariable COX regression analysis using rms package in R, and forest plot was built to visualizing its result. In addition, calibration curve was plotted to show the difference between actual and predicted survival (1-year, 3-year and 5-year respectively). And decision curve analysis (DCA) was used to identify whether this nomogram model was suitable for clinical utility. The x-axis showed the percentage of threshold probability, and y-axis represents the net benefits. Furthermore, ROC curve was used by survival ROC package in R for measuring 1-year, 3-year, and 5-year overall survival to validate the accuracy for survival prediction of Nomogram model.

### Statistical analysis

R (version 4.0.4) and SPSS 22.0 were used for data analysis; The *P*-value less than 0.05 was considered to be statistically significant. Continuous variables were compared between high- and low-risk groups using t testing or one-way ANOVA, while categorical variables were assessed by Fisher’s exact test or the chi-square test. Survival time was calculated from the data of first diagnosis to the data of death. Overall survival (OS) was calculated using Kaplan–Meier method and the log-rank test. Univariable and multivariable Cox proportional hazards regression models were performed to determine independent prognostic factors, including risk score and other clinicopathological features for COAD patients, and the hazard ratio (HR) and its 95%CI were estimated. Time-dependent ROC curve analysis was used to estimate the accuracy of predictive model and prognostic value of gene expression. The prediction was acceptable when the area under the curve (AUC) value was higher than 0.6 (95% CI: 0.5–0.7); it was significant at AUC > 0.7; and it was more predictive when the AUC approached 1^19,20^. The calibration curves and DCA were used to validate the clinical accuracy of predictive model.

### Ethical approval and informed consent

No ethical approval or informed consent was required for this systematic review.

## Results

### Identification of differentially expressed pyroptosis genes

**Figure 1:**
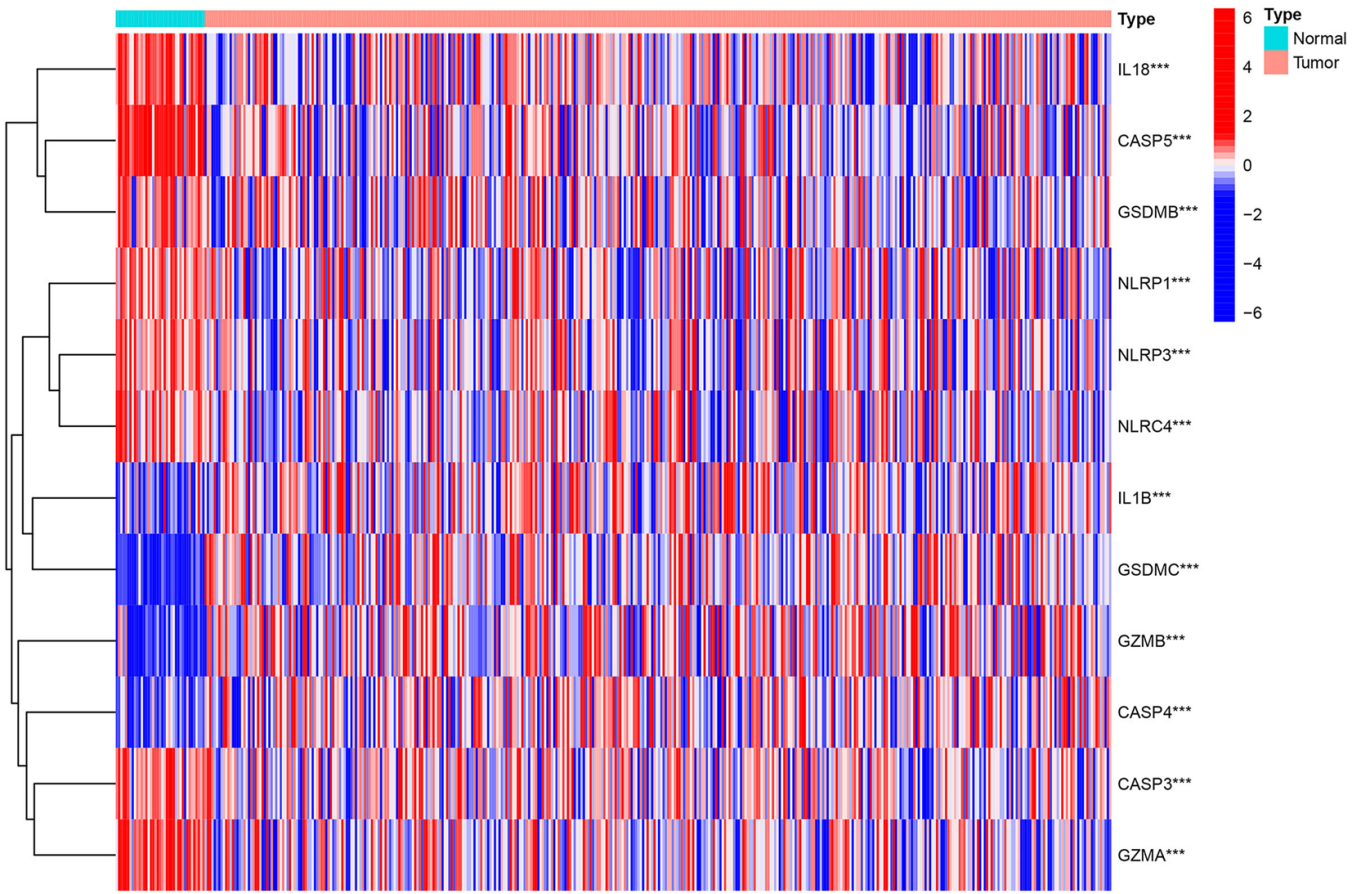
The expression of these differentiated-expressed pyroptosis-related genes.

**Figure 2:**
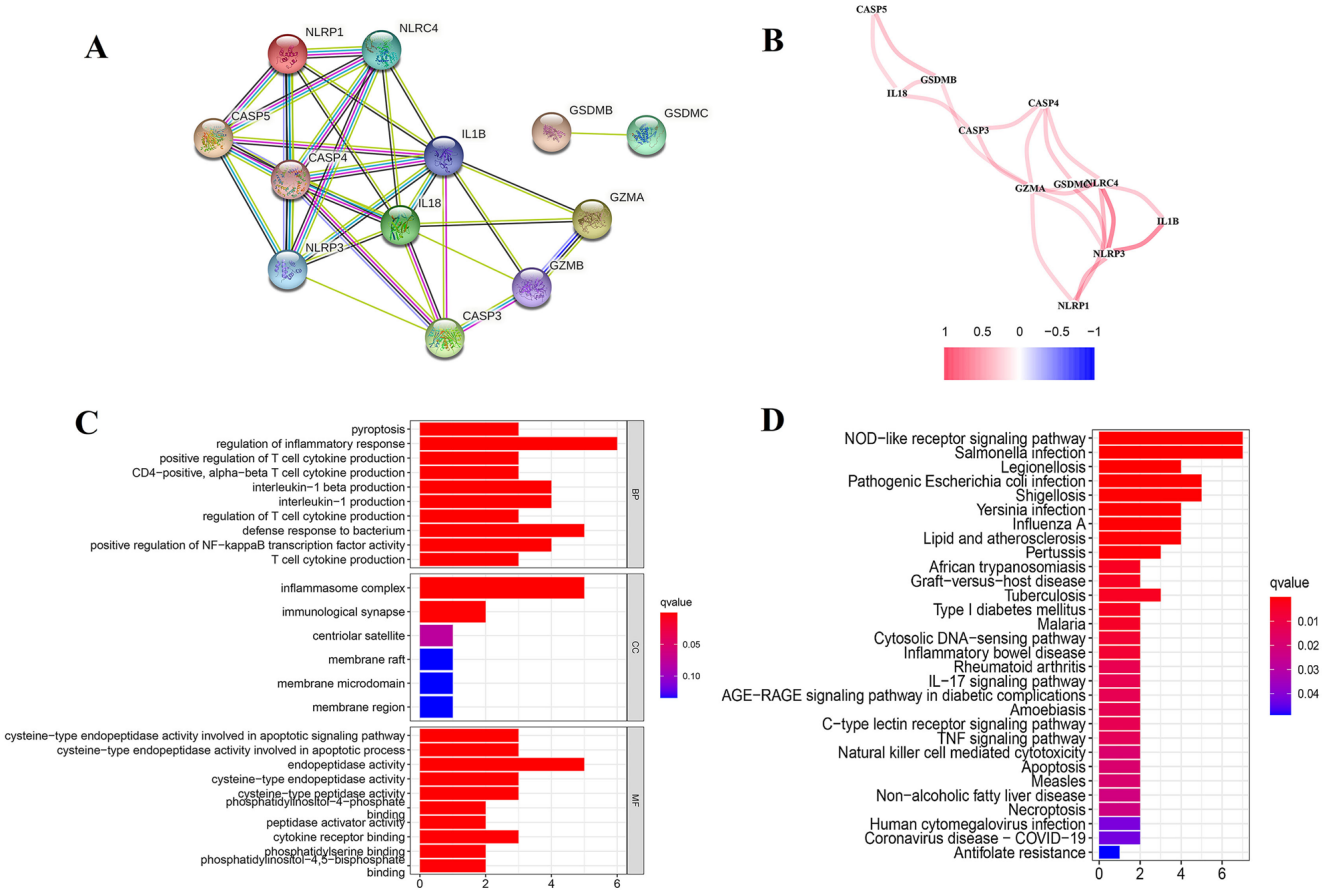
Identification of differentially expressed pyroptosis genes. (**A**) and (**B**) The potential association of biological function and expression level among pyroptosis-related DEGs; (**C**) and (**D**) GO and KEGG analysis for DEGs.

21 pyroptosis-regulated genes were analyzed via searching related articles or studies (genes and their relative articles were listed in Table S1). The mRNA expressed profile and related clinicopathological data was acquired from TCGA-colon adenocarcinoma, including 398 cancer samples and 39 normal samples, and 18 DEGs was included in further analysis. Through the limma package in R, it was found that 12 genes were differentially expressed, in which 8 were down-regulated and 4 were up-regulated significantly in colon adenocarcinoma (*P* value < 0.05) (shown in Table S2). The expression of these differentiated-expressed pyroptosis-related genes was shown as heatmap in Fig. 1. Additionally, the potential association of biological function and expression level among pyroptosis-related DEGs were analyzed by using STRING platform and R respectively (Fig. 2A,B). We found that most of these genes play key roles in some important biological functions, such as building inflammasome complex to regulate cell pyroptosis and apoptosis and promoting the production of T cell cytokine, interleukin-1, NF-kappa transcription factors and other factors related to defense response to mediate immune reaction (Fig. 2C,D). Additionally, these pyroptosis-related DEGs significantly enriched in apoptotic pathway and immune-related pathways, such as, IL-17, TNF signaling pathways, whose association with carcinogenic activation have been proved widely^21–23^. For validating, GSE17526 and GSE39582 were downloaded from GEO database. The data from two microarrays were merged and normalized for further analyzing.

### Identification of classification patterns mediated by 12 differentiated pyroptosis-related genes

**Figure 3:**
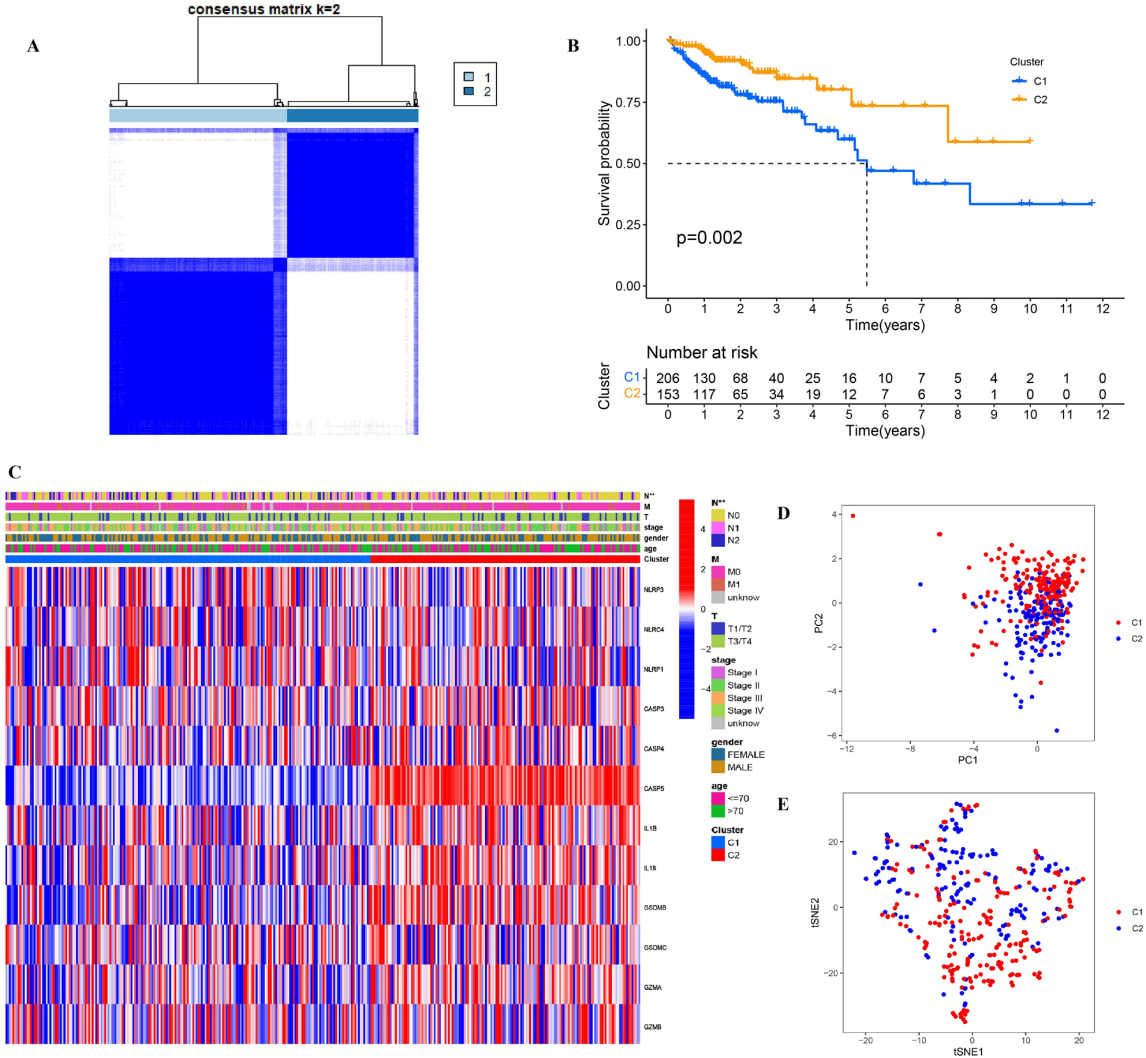
Classification patterns mediated by 12 differentiated pyroptosis-related genes. (**A**) Two different regulation patterns were constructed by using unsupervised clustering method; (**B**) survival difference between clusters; (**C**) clinicopathological characteristics of two clusters. (**D**) and (**E**) PCA analysis for two clusters.

**Figure 4:**
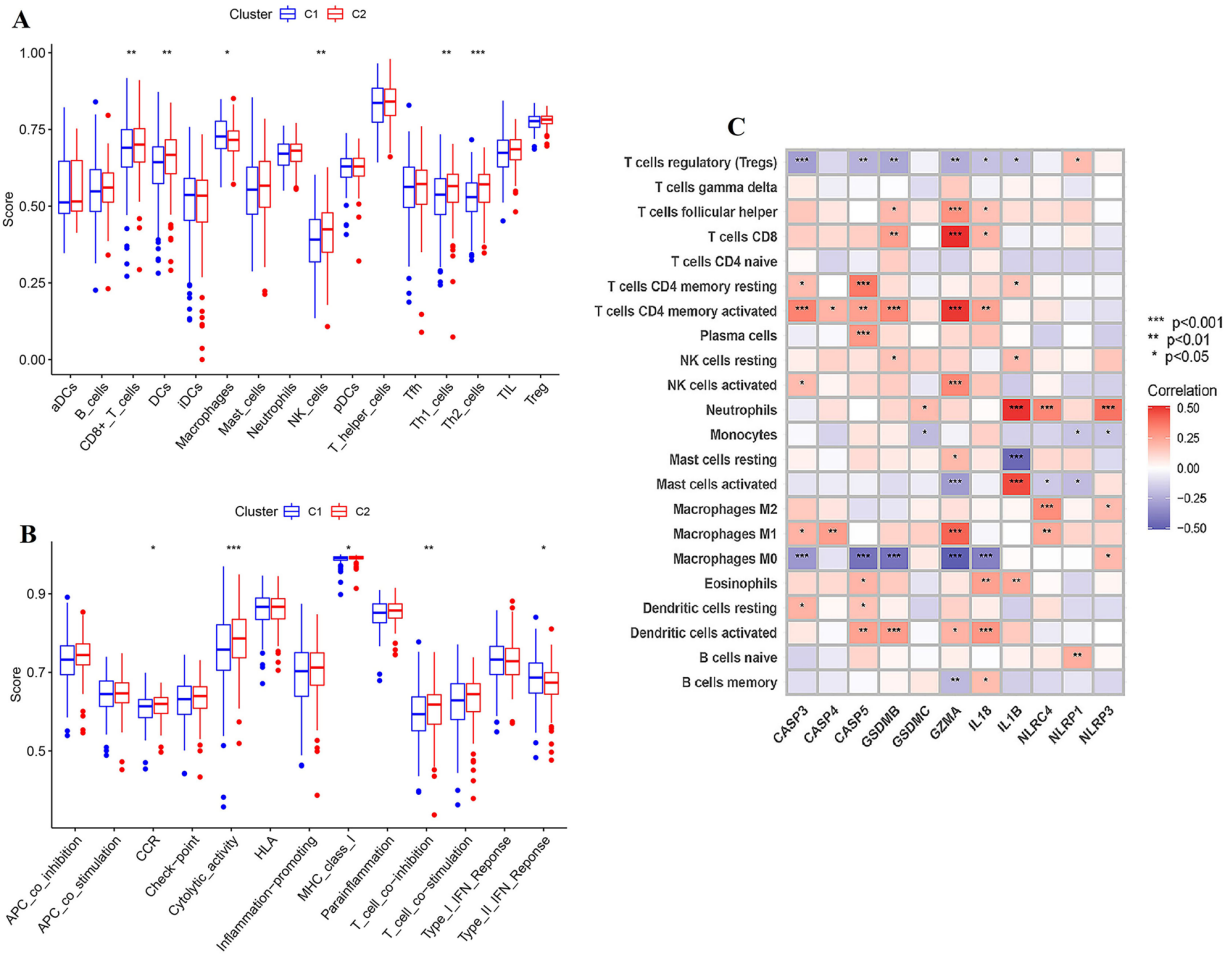
Immune analysis for clusters. (**A**) and (**B**) The difference in immune cell infiltration and immune functions between clusters; (**C**) the correlation between pyroptosis-related genes and infiltration of immune cells.

Two different regulation patterns were constructed by using unsupervised clustering method according to the expression of differentiated-expressed pyroptosis-related genes, including 207 cases in cluster 1 and 153 cases in cluster 2 respectively (Fig. 3A and Table S3). Through survival analysis, we found that patients in C2 showed better prognosis than those in C1 (*P* = 0.002) (Fig. 3B). To explore the difference in clinicopathological characteristics between two clusters, comprehensive study of clinical information was performed and showed as heatmap in Fig. 3C. It can be found that cluster 1 had more advanced N stage, whose association with prognosis of colon adenocarcinoma patients had been proved widely. However, other clinical factors, including M, T stage, pathological stage, gender and age, failed to show the relationship with classification pattern. PCA showed that patients with different clusters were well separated into two groups, which revealed the validity of this classification patterns (Fig. 3D,E). Then, ssGSEA enrichment analysis was performed to explore the differences in activated immune cell infiltration and immune functions between two classification patterns (Fig. 4A,B). Results showed that patients in cluster 2 had more abundant activated innate immune cell infiltration, including CD8 T cells, dendritic cells, macrophages, nature kill cells, as well as helper T cells. Additionally, cluster 2 showed more activated chemokine receptor (CCR) and major histocompatibility complex (MHC), making cells easier to be identified by CD8 T cells, and as a result immune reaction was activated better. And they had higher cytolytic activity, cells in which tend to induce apoptosis or pyroptosis when they were infected. Moreover, targeted therapy was potentially more effective for patients in cluster 2 because of their T cell co-inhibition and positive immune targets. The correlation between pyroptosis-related genes with immune genes was showed in Fig. 4C, which revealed that higher expression of pyroptosis genes was associated with more immune cells infiltration. Considering what has been mentioned above, higher expression of pyroptosis-related genes, more activated immune function and their tendency of PCD and more effective to targeted therapy may be the potential causes of better prognosis of cluster 2 patients.

### Establishment of prognostic gene signature based on pyroptosis-related clusters

**Figure 5:**
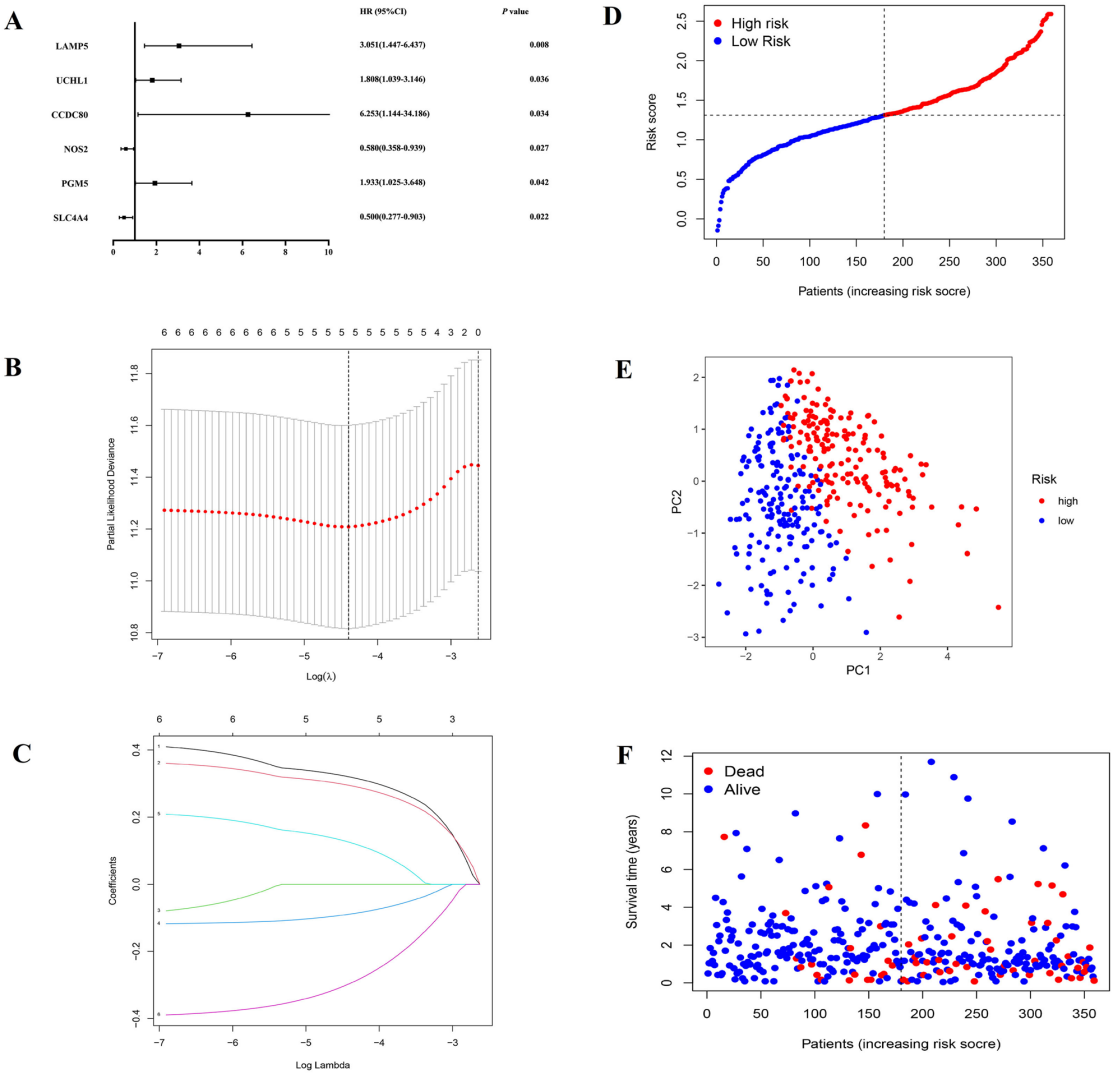
Establishment of prognostic gene signature based on pyroptosis-related clusters. (**A**) Multivariable COX analysis used to identify the survival-related DEGs. (**B**) and (**C**) Pyroptosis-related risk model was constructed by lasso regression. (**D**)–(**F**) Patients in TCGA-CC testing set were divided respectively as low- and high-risk group according to this cut-off value.

To establish a more accurate prognostic pyroptosis-related risk model to predict the survival of colon adenocarcinoma patients, the differences of gene expression between two patterns were explored and a specific gene signature was determined. Based on the quantification of gene signature, risk score was measured for every patient so that it can be applied to the early diagnosis and survival prediction in clinical practice for each patient. Firstly, through limma package in R, it was found that 189 genes were differentially expressed between two patterns, in which 114 were down-regulated and 75 were up-regulated significantly in COAD (|logFC|> 1.0, *P* value < 0.05) (shown in Table S4), and their correlation with pyroptosis-related genes was showed in Fig. S1. Then, through univariable and multivariable COX which were used to identify the survival-related DEGs, six genes were screened in CC (*P* value < 0.05), shown in Figs. S2 and 5A respectively (forest plot with HR and 95% CI). And then, these 6 prognostic-related DEGs were analyzed by LASSO regression to eliminate genes which were closely correlated with other genes. After 1000 resamples, a 5-gene prognostic risk model was constructed (Fig. 5B,C), including lysosomal associated membrane protein family member 5 (LAMP5), ubiquitin C-terminal hydrolase L1 (UCHL1), nitric oxide synthase 2 (NOS2), phosphoglucomutase 5 (PGM5), and solute carrier family 4 member 4 (SLC4A4). The formula used to calculate the risk score was as follows: risk score = LAMP5 expression * 0.319576 + UCHL1 expression * 0.294057 + NOS2 expression * (− 0.088817) + PGM5 expression * 0.124763 + SLC4A4 expression * (− 0.295182). The median risk score of TCGA-COAD cohort was regarded as cut-off value. Patients in TCGA-COAD training set were divided respectively as low- and high-risk groups according to this cut-off value (Fig. 5D). The principal component analysis (PCA) showed that patients with different risks were well separated into two clusters (Fig. 5E). Patients in the low-risk group had lower rate of deaths and a longer survival time than those in the high-risk group (Fig. 5F).

### Performing difference in prognosis and signaling pathways between two subgroups and functional analysis

**Figure 6:**
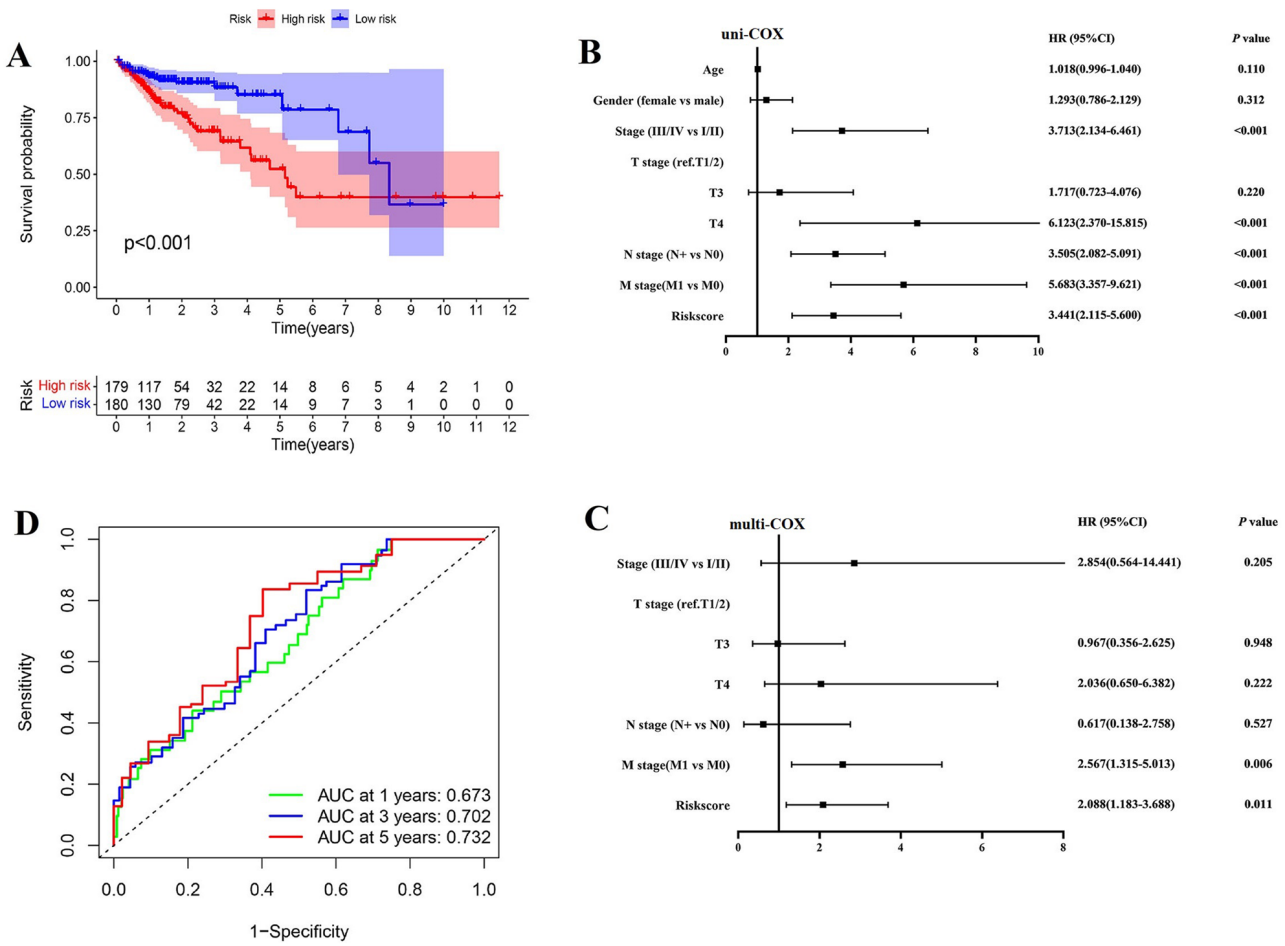

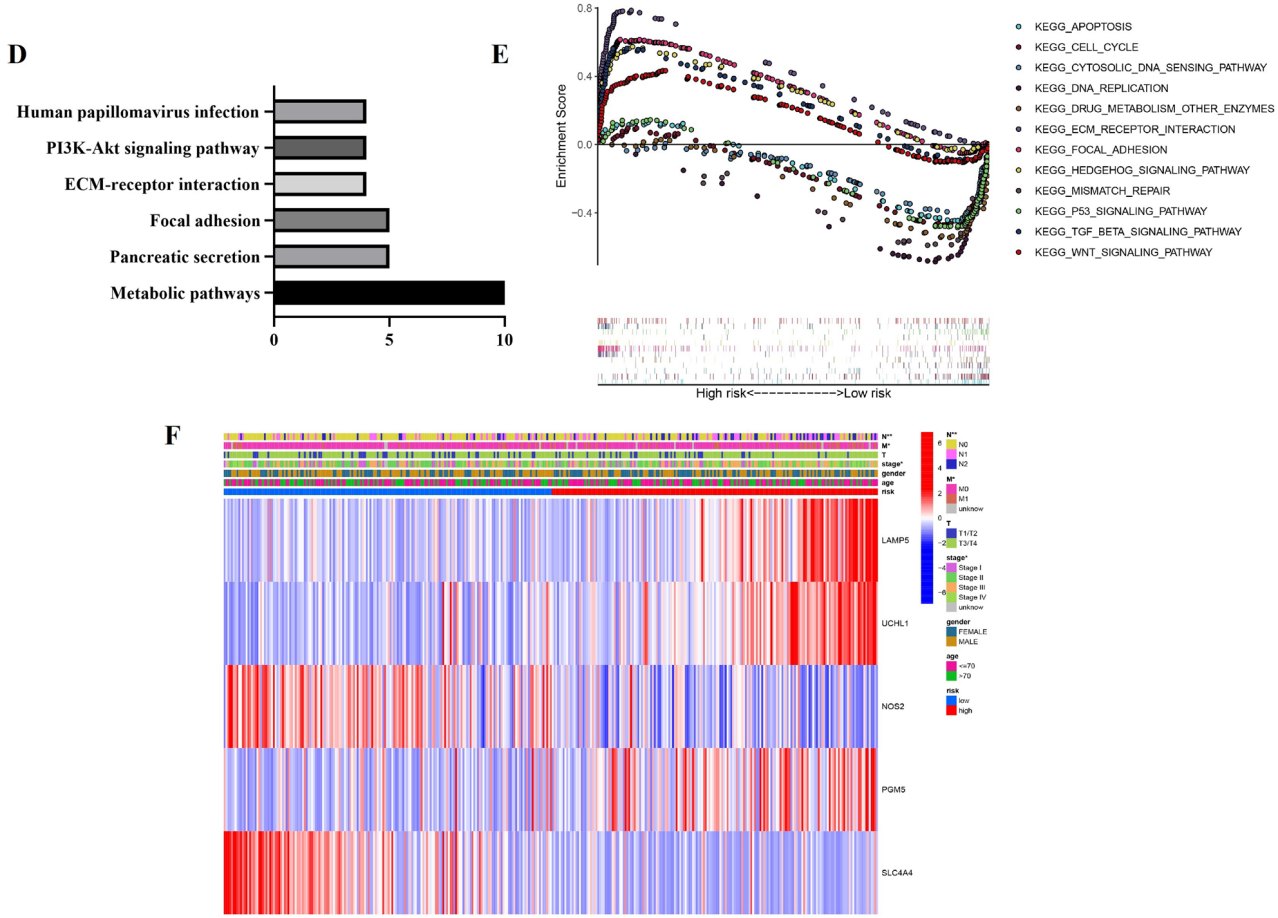
Difference in prognosis and signaling pathways between two subgroups and functional analysis. (**A**) Survival difference between low- and high- risk groups in TCGA training set. (**B**) and (**C**) Univariable and multivariable COX regression including clinicopathological traits and riskscore for OS. (**D**) ROC analysis to estimate the predictive efficiency of risk model. (**E**) KEGG functional analyses based on the risk model. (**F**) GSEA performed to show the difference in signaling pathway between subgroups. (**G**) clinical characteristics of patients.

Survival difference between two groups showed that colon adenocarcinoma patients with lower risk had better prognosis than those with higher risk (*P* < 0.001 in TCGA training set (Fig. 6A). To estimate whether the prognostic model can be independently predictive for survival of colon adenocarcinoma patients, univariable and multivariable COX regression including clinicopathological traits and riskscore for OS were performed. Results showed M stage (HR: 2.567, 95% CI: 1.315–5.013, *P* = 0.006), and risk score (HR: 2.088, 95% CI: 1.183–3.688, *P* = 0.011), were significantly associated with OS (Fig. 6B,C). Furthermore, based on the risk score and clinical traits, we estimate the predictive efficiency of the prognostic model with respect to OS of 1, 3 and 5 year respectively (Fig. 6D). It showed that the area under the ROC curve values were 0.673 at 1 year (95% CI: 58.05–76.54), 0.702 at 3 years (95% CI: 60.82–79.60) and 0.732 at 5 years (95% CI: 65.71–80.72). Thus, the prognostic model presented better predictive accuracy for long-term survival. KEGG functional analysis based on the risk model was performed to explore the enrichment in pathways between two subgroups. We found that DEGs between two risk groups enriched in tumorigenesis, including PI3K-Akt signaling pathway and ECM-receptor interaction pathway, and infection and metabolic diseases-related pathway (Fig. 6E). GSEA performed to show the difference in signaling pathway between subgroups (Fig. 6F). It can be concluded that low-risk groups showed activity in metabolic and self-repair-related pathways, such as drug metabolism, DNA replication, apoptosis, cytosolic and sensing pathway, and better promotion of normal physiological processes, especially cell cycle. Additionally, low-risk group had higher expression of anti-tumor activity, such as activation of P53 pathway. However, some pro-tumor pathways have been expressed in high-risk group, such as TGF-β signaling, WNT signaling, ECM, and Hedgehog signaling pathways, and more activated local adhesion can be seen which potentially led to tumor metastasis. Thus, patients in high-risk group showed bad prognosis because of higher expression of tumorigenesis or metastasis-related pathways, while those in low-risk group had longer lifetime mainly resulted from their good at self-repair and promotion of normal physiological process and anti-tumor characteristics. Clinical analyses showed that patients in high-risk groups had more advanced N stage, larger proportion of metastasis and later pathological stage (Fig. 6G). Thus, this risk model can better predict the survival of colon adenocarcinoma patients, high-risk group of which showed poorer prognosis potentially was caused by more activated tumor-related pathways and their tendency of lymph node and distant metastasis and advanced pathological TNM stages.

### Validating the pyroptosis-related regulators signature

**Figure 7:**
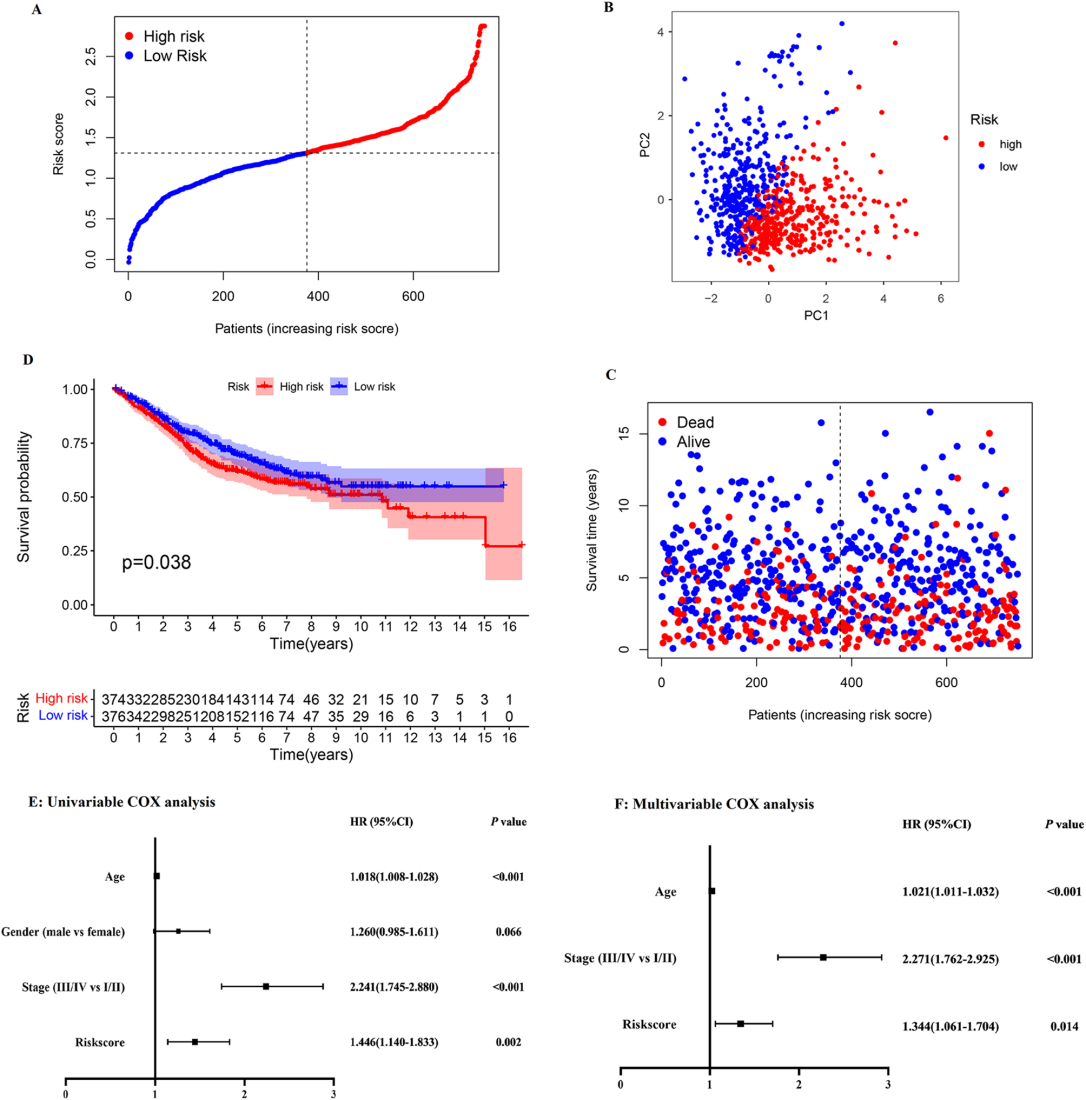
Validating the pyroptosis-related regulators signature. (**A**)–(**C**) Patients in GEO validating set were divided into low- and high- risk groups, and PCA and survival difference between two clusters have been showed. (**D**) KM analysis for GEO cohort to explore the survival difference between groups. (**E**) and (**F**) Univariable and multivariable COX analysis including clinical traits and risk score of patients in GEO validating set.

Colon adenocarcinoma patients in validating sets downloaded from GEO were divided into low- and high- risk groups as mentioned above, PCA and survival difference between two clusters have been showed, results of which have presented that the patients in high-risk group had higher rate of death (Fig. 7A–C). KM survival analysis demonstrated that low-risk group showed longer survival (*P* = 0.038) (Fig. 7D). Thus, this five-DEGs based prognostic model is significantly predictive for survival of COAD patients. Then, univariable and multivariable COX analysis including clinical traits and risk score of patients in GEO validating set were performed. The results showed continues risk score can be independently associated with OS (HR:1.344, 95% CI: 1.061–1.704, *P* = 0.014) which was consistent with the result of TCGA training set (Fig. 7E,F). Though repeated validation in training and validating datasets, it has been proved that this prognostic model can be potentially accessible for predicting the survival of colon patients.

### Construction of a nomogram

**Figure 8:**
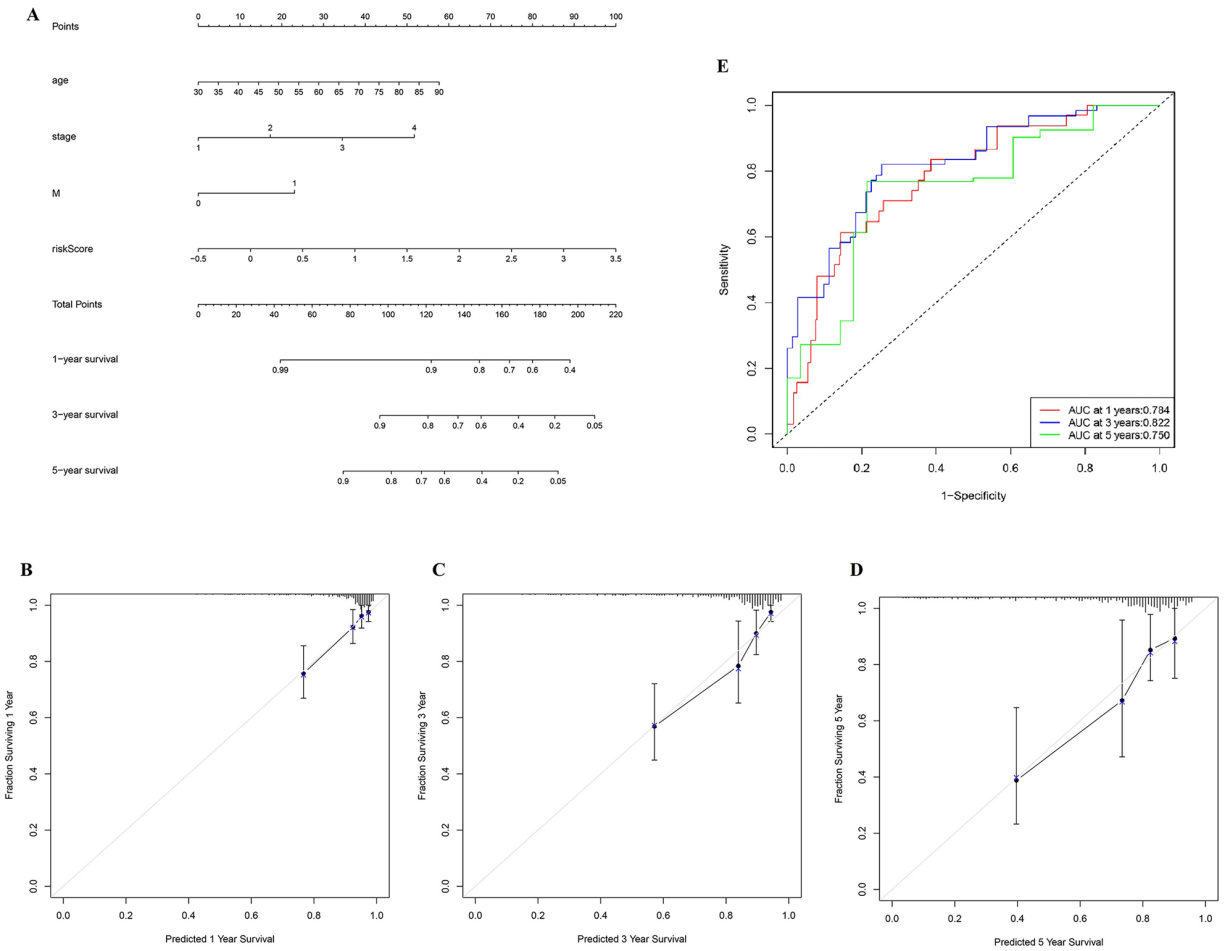
Construction of a nomogram. (**A**) A nomogram model was constructed. (**B**)–(**D**) Calibration plots showed the nomogram model had a similar performance when compared to ideal model. (**E**) ROC curves predict the predictive ability of our model.

Based on the COX regression analysis and clinical practice, we constructed a nomogram model including three prognostic factors, namely age, pathological stage, M stage, and pyroptosis-related regulators signature. Through nomogram model, clinicians can predict the 1-year, 3-year, and 5-year survival probability with quantitative method (Fig. 8A). Patients would receive a points for each parameter, and those with higher total point always suffered from poorer prognosis. The calibration plots showed our nomogram model had a similar performance when compared to ideal model (Fig. 8B–D). Notably, ROC curves predict the predictive ability of our model. AUC values were 0.784 at 1 year (95% CI: 70.16–86.63), 0.822 at 3 year (95% CI: 74.32–90.06) and 0.750 at 5 year (95% CI: 62.12–87.47), thus our nomogram is ideal to predict the prognosis of colon adenocarcinoma patients (Fig. 8E). Additionally, DCA curve revealed that the nomogram model was better at predicting prognosis and was more applicable to clinical utility than any other factors (Fig. S3).

### Comparison of the difference in immune activity between two risk groups

**Figure 9:**
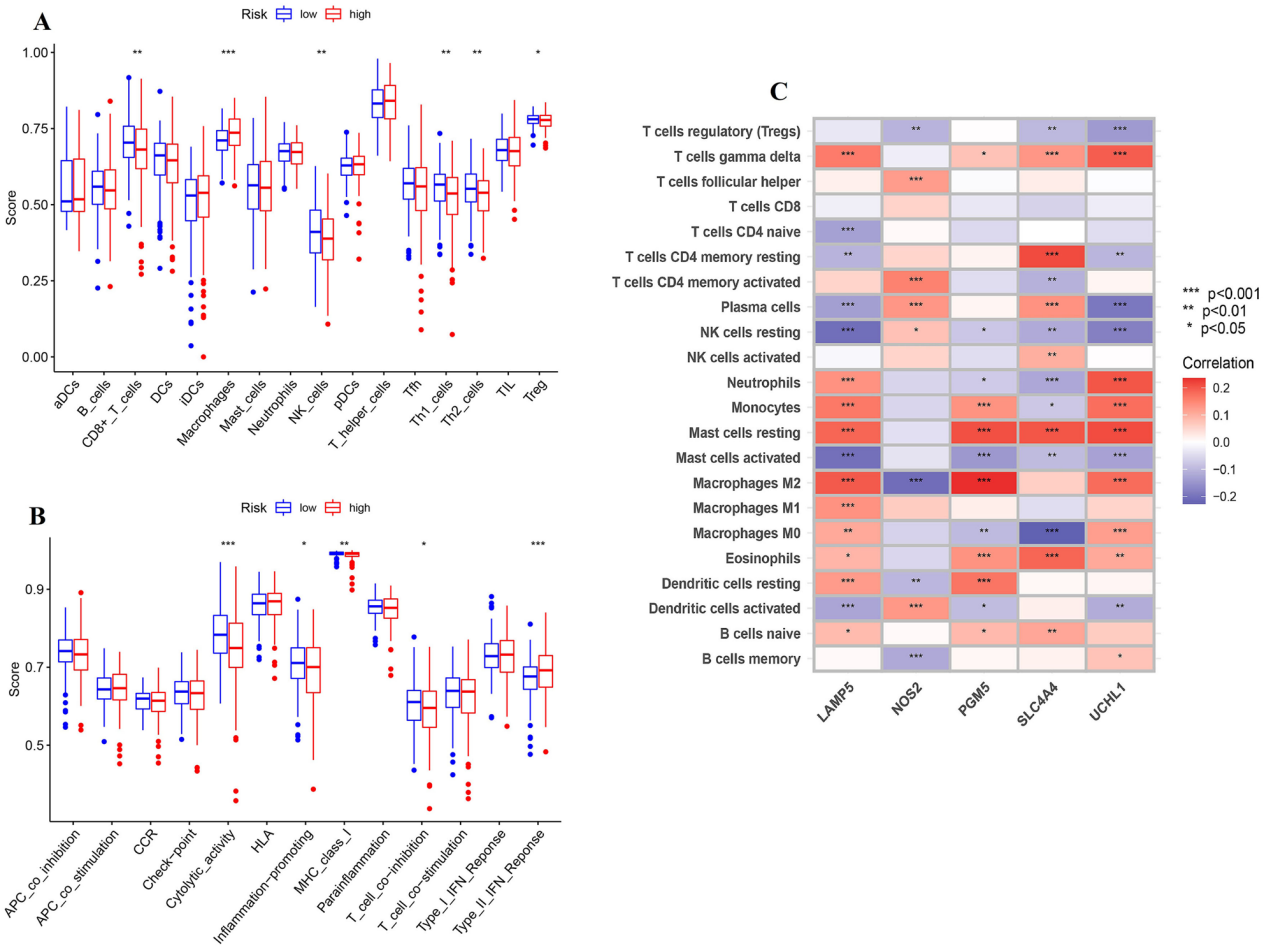
Comparison of the difference in immune activity between two risk groups.

**Figure 10:**
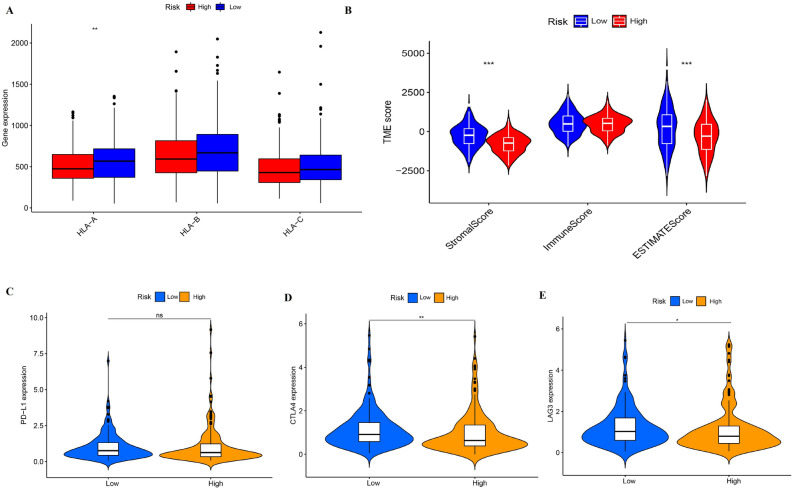
Immune-related analysis between groups. (**A**) The expression difference of HLA family between groups. (**B**) Immune score to estimate tumor purity for groups. (**C**)–(**E**) The expression of typical immune checkpoints.

**Figure 11:**
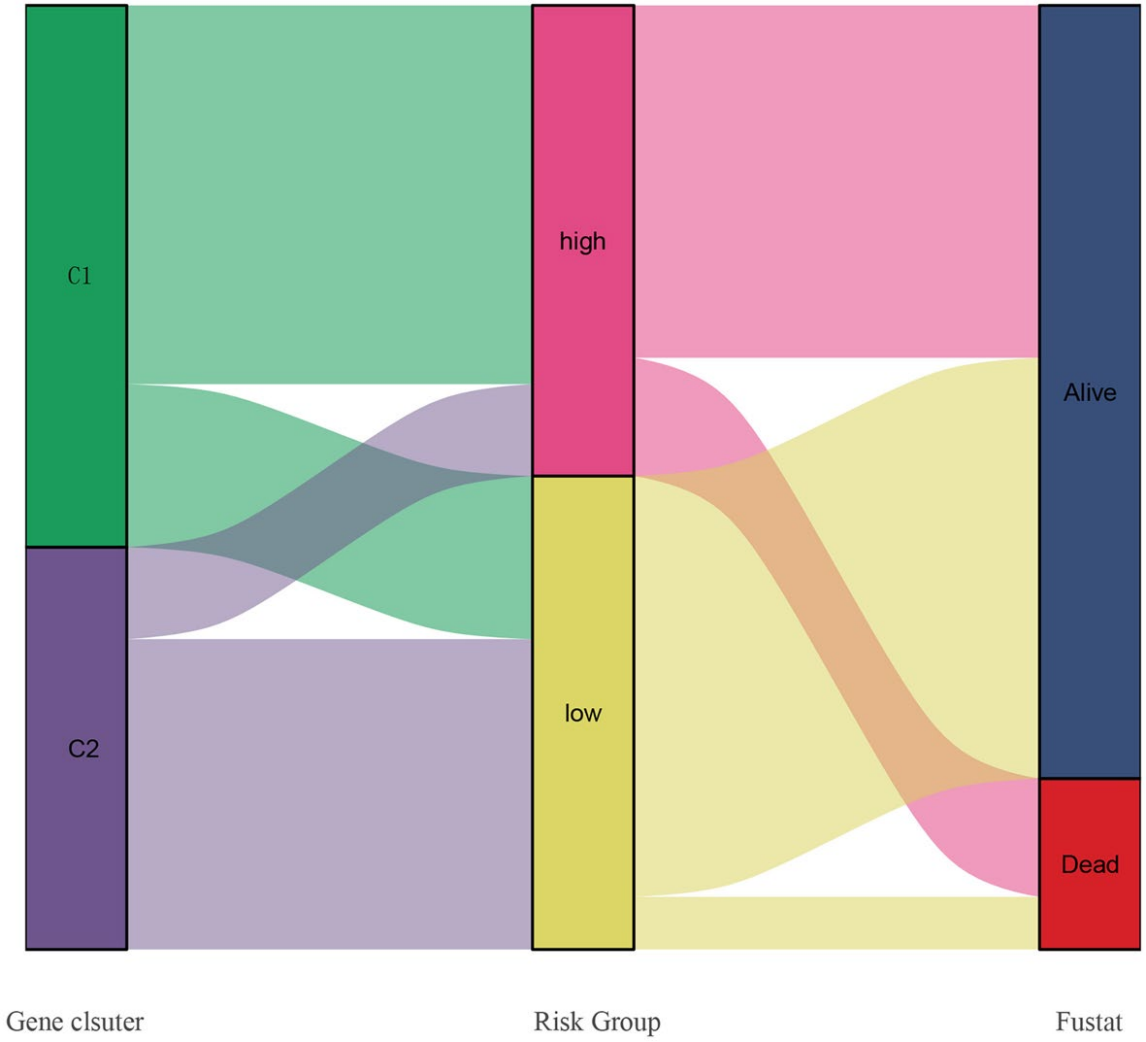
Sankey diagram was performed to present the relationship among clusters, risk groups and prognosis.

We perform the ssGSEA enrichment analysis to explore the differences in activated immune cell infiltration and immune functions between subgroups (Fig. 9A,B), and the correlation between gene signature and infiltrated immune cell was presented in Fig. 9C. It can be concluded that low-risk group had more immune cell infiltration, especially CD8 T cell, NK cells, and helper T cells which were abundant in tumor microenvironment and important for the induction of immune activities. Tumor-associated macrophages (TAM) played a dual role in the process of carcinogenesis and tumor metastasis, including anti-tumor function of M1-type TAM and pro-tumor function of M2-type TAM. Thus, higher expression of macrophages in high-risk group may lead to growth and development of tumor because of their pro-tumor function. Additionally, it had been showed that some inflammation- or immune- related pathways were activated in low-risk group, such as cytolytic activity, inflammation-promoting pathway, and activated MHC. Notably, HLA-A was significantly highly expressed in low-risk group, which illustrated that low-risk tumors could activate immunity against tumor development to larger extent (Fig. 10A). Then, although immune scores of two groups were similar, patients in low-risk group had higher stroma score and estimate score (Fig. 10B). According to previous results, immune cells playing central roles in the immunity induction, such as CD8 T cell, NK cells, and helper T cells, were more abundant in low-risk group. Hence, it was concluded that low-risk tumors presented more stromal cells, lower tumor purity and more activated immunity than high-risk ones. Furthermore, the difference of immune checkpoints between groups was compared. The significant overexpression of CTLA4 and LAG3 in low-risk patients predicted that those patients were more beneficial to anti-CTLA4 and anti-LAG3 targeted treatment (Fig. 10C–E). Additionally, gene signature was closely associated with infiltration of immune cells. It can be concluded that more immune cells infiltrating and more activated immune- or inflammation- related pathways as well as higher tumor purity may be the main causes of better prognosis of colon patients in low-risk group, and low-risk patients should be suggested to anti-CTLA4 and anti-LAG3 targeted treatment. Lastly, Sankey diagram was performed to present the relationship among clusters, risk groups and prognosis (Fig. 11).

## Discussion

As the leading cause of cancer-related death, the poor prognosis of COAD patients is mostly contributed to their late diagnosis, and high risk of recurrence and metastasis^3,24^. Early diagnosis is prominent in improving the survival of COAD patients. Tumor markers are stably expressed in fluid or tissue with disease-specific characteristic, and they can potentially indicate the occurrence of cancer in the early stage. Based on the previous studies, numerous tumor markers and their functional mechanism have been validated, but mostly were focused on one single gene, thus resulting in the limited clinical significance. To improve the predictive significance, a novel multiple pyroptosis-related gene model was built to predict the survival of COAD patients at the first time.

Pyroptosis, as a type of PCD, induced inflammation in vivo and resulted from pole formation, cell swelling and rupture, and then released the cell content. Pyroptosis may play pro-tumor or anti-tumor factors for the dual roles of inflammation in the process of carcinogenesis and tumor metastasis^11,14,25^. On the one hand, inflammasome can eliminate the microbes and keep the integrity of intestinal epithelial, aiming to protect against cancer attracts^11^. On the other hand, inflammasomes can stimulate the production of trophic factors for tumor cells and IL-18 and IL-1β can recruit immune suppressive factors and impair the functions of natured kill cells, thus making tissue more susceptible to tumor^13^. As a result, inconsistent conclusions highlighted that pro-tumorigenic and anti-tumorigenic properties are mostly determined by the kinds of diseases and cells. For example, it has been reported that only sustained circuit of IL-18 in lung cancer, rather than contemporary exposure, can potentially cause anti-tumor immunosurveillance and further inhibit tumor growth and metastasis. As for gastrointestinal cancers, the causes were mostly associated with the infection of microbes. Meanwhile, the anti-tumor signature of pyroptosis has been widely proved, potentially due to the protective function of inflammasome to gastrointestinal epithelial. Therefore, it can be concluded that pyroptosis can inhibit the growth and development of colon adenocarcinoma^11^. However, it is still unclear how to use pyroptosis to improve the early diagnosis and predict the survival of COAD patients in clinical practice. In this study, a risk model was constructed based on the expression of five pyroptosis-related regulators. It has been proved that this risk model was independently associated with the prognosis of patients, and those in low-risk group had better prognosis than those with high risk.

In this study, the transcriptome data and related clinicopathological data were downloaded from TCGA and GEO databases, and the differentially expressed pyroptosis-related genes were screened. According to the expression of these 8 DEGs, patients were grouped into two clusters. COAD patients in cluster 2 had better prognosis than those in cluster 1, which may result from their larger proportion of immune cells infiltration and more activated immune function. Moreover, clinical analysis revealed that C1 patients had more advanced N stage, and thus they had larger risk of tumor metastasis. Considering that only 21 pyroptosis-related genes were included in our study through searching online for the limited studies, the different expressed genes between two clusters were further screened and constructed based on 8 different-expressed pyroptosis-related genes to increase the predictive efficacy of risk model. Through Cox and Lasso regression analysis, five genes were screened, including LAMP5, UCHL1, NOS2, PGM5, and SLC4A4.

LAMP5, a member of lysosome-associated membrane protein family, was upregulated in various kinds of tumor, such as colon adenocarcinoma and acute leukemia. LAMP5 was associated with some tumor-related signaling pathways, especially NF-KB pathway, as well as the inhibition of the activity of Toll-like receptor or IL-1 receptor^26,27^. Some studies proved that the dysregulation of LAMP5 was related to the infiltration of tumor immune cells and associated with the poorer prognosis of COAD patients^27,28^. UCHL1, a member of deubiquitinating enzymes, plays a key role in tumor growth and metastasis. Growing evidence illustrated that the methylation status and deubiquitinating activity of UCHL1 were associated with patients’ prognosis^29–31^, and its higher expression in tumor activated the Wnt-β-catenin/TCF pathway to promote cell proliferation, migration and invasion^30^. Moreover, clinical studies displayed that the patients with higher expression of UCHL1 had lager tumor size and more advanced stage and more metastatic lymph nodes^31,32^, which is consistent with the results of our study. Its deubiquitinating activity was related to cell death, potentially inducing cell pyroptosis and apoptosis, whereas more studies are needed to explore its association with pyroptosis. NOS2 was upregulated by pro-inflammatory-stimulated macrophages and tumor hypoxia environment, accounting for the production of NO, thereby resulting in tumor cell death via increasing the stabilization of tumor suppressor P53 and promoting the induction of IL-6 and IL-8. At the same time, NOS2 was downregulated in colon tumor and associated with poor prognosis of COAD patients^33–35^. It can be concluded that NOS2 was important for the induction of immune reaction and could lead to cell death. PGM5 was the key enzyme in the glucose metabolism, and its low expression in colon adenocarcinoma was important to regulate the development and invasion of tumor. Some studies revealed that COAD patients with increased expression of PGM5 had better prognosis^36–38^. However, in our study, PGM5 was upregulated in tumor, which was potentially related to its key function in aerobic metabolism that was strongly activated in the process of tumor growth and metastasis. More studies are needed in the future. As a bicarbonate transporting proteins responsible for maintain PH equilibrium in the cells, SLC4A4 was downregulated in the colon adenocarcinoma and suppressed tumor development and metastasis. Its association with the prognosis of COAD patients has been widely proved^24,39^. Additionally, recent studies discovered that cells with higher expression of SLC4A4 had more immune cell infiltration, and it was potentially seen as the promising immune-related therapeutic target^40^. Thus, SLC4A4 may suppress tumor by stimulating immune system, and, as a result, more immune cells in tumor microenvironment were activated to fight the cancer. It can be concluded that these genes related to the activation of immune system or regulation of cell death may play targeted protein or key molecular role in the pathway of cell pyroptosis, thus further inducing tumorigenesis and development. More studies are needed in the future.

The risk model based on the expression of five pyroptosis-related genes mentioned above can predive the prognosis of COAD patients independently. Multivariable COX regression, including clinicopathological traits and risk score, revealed that risk score was independently associated with OS for colon adenocarcinoma patients (*P* = 0.011 in TCGA training set; *P* = 0.014 in GEO validating set). When further validating this predictive model in ROC curve, it showed that risk score performed better predictive efficiency when predicting the longer survival of COAD patients. In clinicopathological analysis, it was obvious that high-risk group was associated with more advanced N stage, larger proportion of distant metastasis, and later TNM stage.

In this study, ssGSEA enrichment analysis and ESIMATE were carried out to explore the differences in immune cell infiltration and immune functions between difference risk groups. Interestingly, it found that low-risk groups had more infiltrated immune cells, such as CD8 T cell, NK cells, and helper T cells, and more activated immune pathways, including cytolytic activity, inflammation-promoting pathway, and activated MHC, as well as highly expressed HLA-A and some promising targeted protein for target therapy or immune therapy, including CTLA4 and LAG3. Moreover, our study showed that high-risk group had less stromal cells and higher tumor purity in TME. Additionally, GSEA analysis demonstrate that the low-risk group mainly enriched in some metabolic and self-repair-related pathways was conducive to maintain the balance of Internal environment and homeostasis, while the high-risk group was enriched in some pro-tumor signaling pathways, such as TGF-β, WNT, and ECM signaling pathways, thus leading to the tumor growth and metastasis^23,41–43^. Therefore, inhibited immune and inflammation system as well as widely activated tumor-related pathways in high-risk group may be the reasons of their poorer prognosis. Anti-CTLA4 and anti-LAG3 targeted therapy should be suggested to low-risk patients.

Additionally, a nomogram model was constructed in this study based on prognostic signature and clinical factors, including age, pathological TNM stage, M stage and risk score. The best AUC was observed in combined nomogram, instead of one single factor, in which the risk score had promising prognostic signature. It has proved that nomogram performed better when predicting for longer survival of COAD patients. The combined prognostic signature and clinicopathological factors comprehensively defined the pathogenetic mechanism and phenotype for colon adenocarcinoma, which deserves to be evaluated in future studies.

Pyroptosis was strongly associated with tumor for its induction of inflammation and greatly activating immune system, thereby further resulting in cell death. Especially for patients with colitis-associated colon adenocarcinoma, cell pyroptosis was initiated rapidly by the invasion of microbes, as tumor-inhibiting process suppressed tumor development and metastasis, thus improving patients’ prognosis, which has been proved in our study. Compared with other risk models, most of which are based on hypoxia, m6A methylation, ferroptosis, and immunity, pyroptosis can directly cause cell death. Notably, pyroptosis can change tumor microenvironment and further decide tumor development. Additionally, genes included in our model also play prominent roles in tumor-related signaling pathways, immune cell infiltration, gene ubiquitylation and methylation, inflammation, glucose and aerobic metabolism, causing cell pyroptosis eventually. Thus, pyroptosis can be deemed as an outcome of complex tumor progressing and pyroptosis-based model is more valuable in predicting prognosis and improving treatment effect.

However, pyroptosis-related studies were far from enough, especially for Chinese patients. It was well-known that gut microbiota may be different among various races, which contributes to the varied role of inflammasome in the process of tumorigenesis. Consequently, more studies are needed to explore the association between pyroptosis and tumor for Chinses patients. Additionally, most chemotherapy drugs were worked by inducing apoptosis, but failed at the end, because drug resistance developed. Pyroptosis may be a promising way to improve the effects of chemotherapy and it deserves more studies in the future^2,44^.

## Conclusion

Based on five pyroptosis-related genes signature and being constructed in this study, the risk model can be predictive for the survival of COAD patients and beneficial for improving the individual treatment and increasing the accuracy of early diagnosis. That patients with higher risk had poorer prognosis were associated with more advanced tumor stage and higher risk of metastasis, and resulted from highly activated pro-tumor pathways and inhibited immune system and poorer integrity of intestinal epithelial. This study proved the relationship between pyroptosis and immune, which offered basis for future studies. In the follow-up experiment, its predictive efficiency in clinical practice based on Chinese population will be verified.

## Supplementary Information

Supplementary Figure S1.

Supplementary Figure S2.

Supplementary Figure S3.

Supplementary Table S1.

Supplementary Table S2.

Supplementary Table S3.

Supplementary Table S4.

## Data Availability

All data, including human-related ones, was deposited in public datasets TCGA and GEO (GSE17526 and GSE39582), which were publicly available. R (version 4.0.4) and SPSS 22.0 were used for data analysis, and related packages and codes were displayed in manuscript. Please contact Baojun Huang, whose E-mail is bjhuang@cmu.edu.cn, if someone wants to request the data from this study.
