# Supplementary figures and images for: Pyroptosis is related to immune infiltration and predictive for survival of colon adenocarcinoma patients
Source: Sci Rep. 2022 Jun 2;12:9233. doi: 10.1038/s41598-022-13212-2 (PMC9163148; doi:10.1038/s41598-022-13212-2)

Figure S1: The correlation between DEGs and pyroptosis-related genes

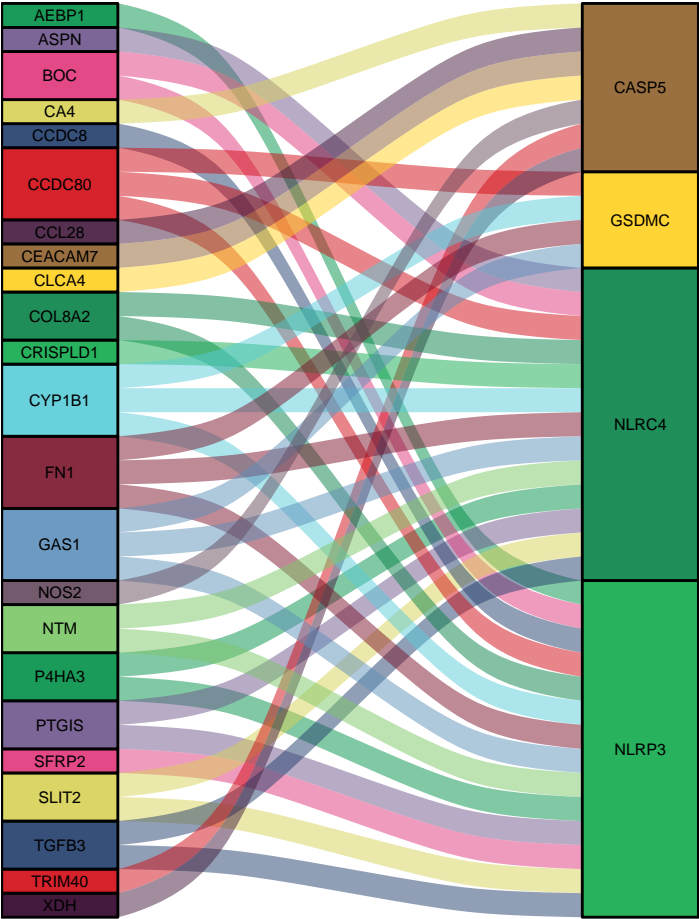

DEGs

Pyroptosis Genes

Supplement: Supplementary file 1 — Supplementary Figure S1. [file 41598_2022_13212_MOESM1_ESM.pdf]

Figure S2: univariant COX used to identify the survival-related DEGs

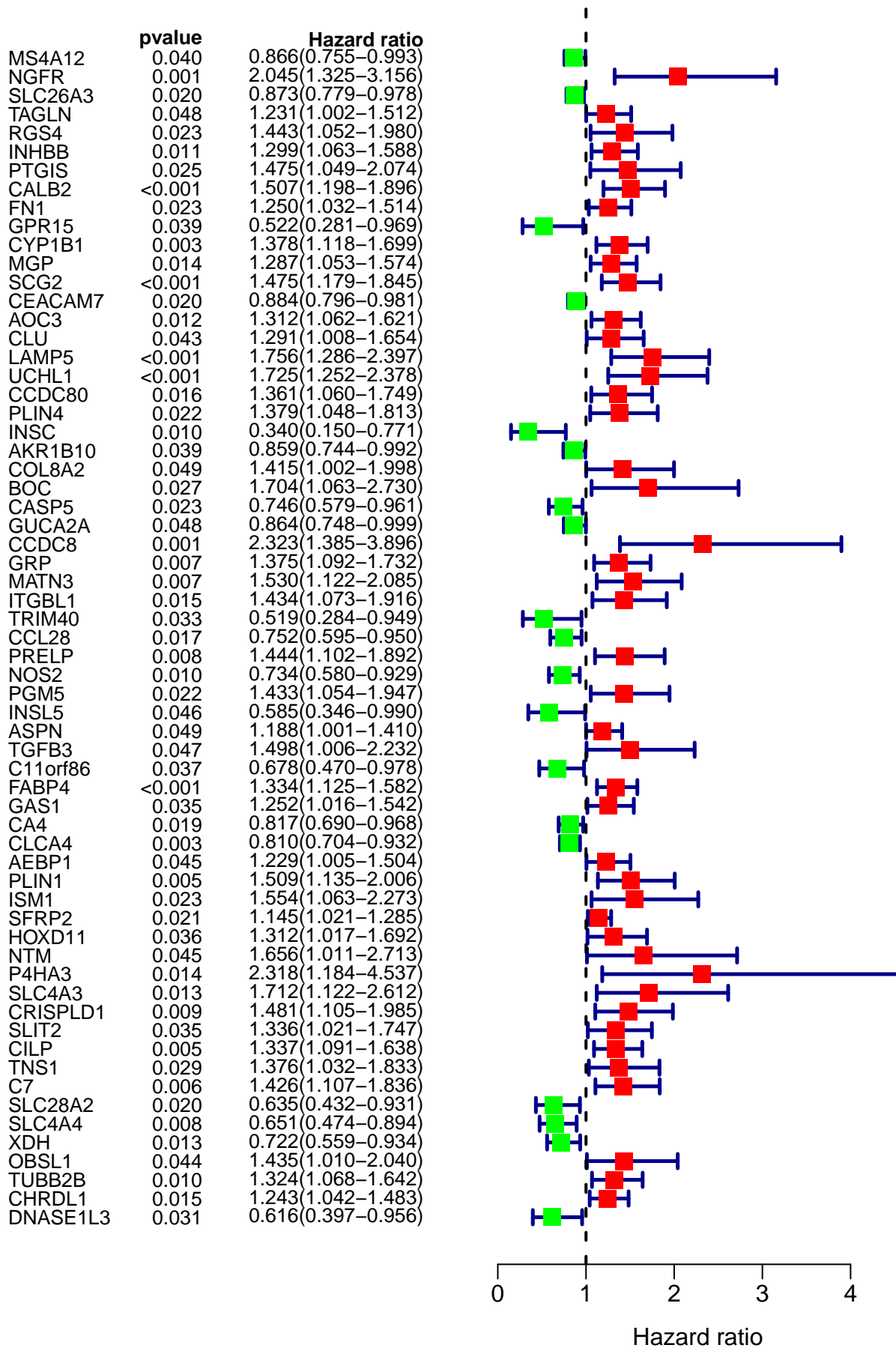

Supplement: Supplementary file 2 — Supplementary Figure S2. [file 41598_2022_13212_MOESM2_ESM.pdf]

Figure S3: DCA curve for nomogram model

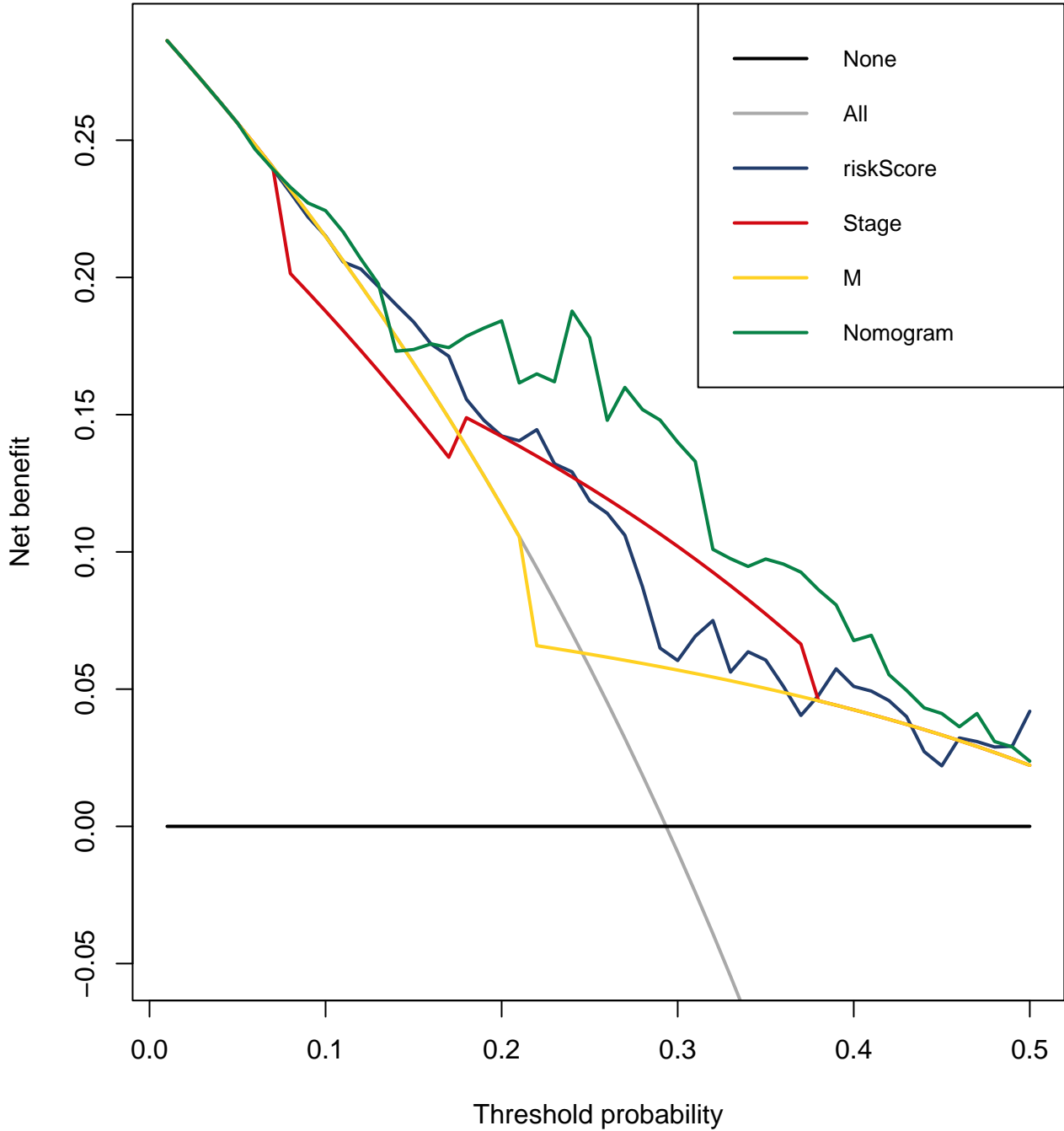

Supplement: Supplementary file 3 — Supplementary Figure S3. [file 41598_2022_13212_MOESM3_ESM.pdf]
