# Supplementary material for: Pyroptosis is related to immune infiltration and predictive for survival of colon adenocarcinoma patients
Source: Sci Rep. 2022 Jun 2;12:9233. doi: 10.1038/s41598-022-13212-2 (PMC9163148; doi:10.1038/s41598-022-13212-2)
Supplement: Supplementary file 4 — Supplementary Table S1. [file 41598_2022_13212_MOESM4_ESM.docx]

Table S1: Pyroptosis-related genes

NLRP1: 1-3

NLRP12: 4,5

NLRP3: 3,6-8

NLRC4: 3,5,9

NLRP6: 10,11

AIM2: 3,12

LXRs: 13

NALP1: 14,15

CASP1: 16,17

CASP3: 18-20

CASP4: 8,21,22

CASP5: 8,22

CASP11: 21,23

IL1B: 22,24,25

IL18: 22,24

GSDMB: 26-28

GSDMC: 28,29

GSDMD: 21,24,25,30

GSDME: 19,20

GZMA: 27

GZMB: 31

Reference

1 Yan, J. et al. CCR5 Activation Promotes NLRP1-Dependent Neuronal Pyroptosis via CCR5/PKA/CREB Pathway After Intracerebral Hemorrhage. Stroke 52, 4021-4032, doi:10.1161/strokeaha.120.033285 (2021).

2 Taabazuing, C. Y., Griswold, A. R. & Bachovchin, D. A. The NLRP1 and CARD8 inflammasomes. Immunological reviews 297, 13-25, doi:10.1111/imr.12884 (2020).

3 Guo, Q. et al. Cytokine Secretion and Pyroptosis of Thyroid Follicular Cells Mediated by Enhanced NLRP3, NLRP1, NLRC4, and AIM2 Inflammasomes Are Associated With Autoimmune Thyroiditis. Frontiers in immunology 9, 1197, doi:10.3389/fimmu.2018.01197 (2018).

4 Chen, H. et al. NLRP12 collaborates with NLRP3 and NLRC4 to promote pyroptosis inducing ganglion cell death of acute glaucoma. Molecular neurodegeneration 15, 26, doi:10.1186/s13024-020-00372-w (2020).

5 Chen, H. et al. NLRP12- and NLRC4-mediated corneal epithelial pyroptosis is driven by GSDMD cleavage accompanied by IL-33 processing in dry eye. The ocular surface 18, 783-794, doi:10.1016/j.jtos.2020.07.001 (2020).

6 Qiu, Z. et al. Lipopolysaccharide (LPS) Aggravates High Glucose- and Hypoxia/Reoxygenation-Induced Injury through Activating ROS-Dependent NLRP3 Inflammasome-Mediated Pyroptosis in H9C2 Cardiomyocytes. Journal of diabetes research 2019, 8151836, doi:10.1155/2019/8151836 (2019).

7 Liang, Q. et al. Lycorine ameliorates bleomycin-induced pulmonary fibrosis via inhibiting NLRP3 inflammasome activation and pyroptosis. Pharmacological research 158, 104884, doi:10.1016/j.phrs.2020.104884 (2020).

8 Baker, P. J. et al. NLRP3 inflammasome activation downstream of cytoplasmic LPS recognition by both caspase-4 and caspase-5. European journal of immunology 45, 2918-2926, doi:10.1002/eji.201545655 (2015).

9 Sundaram, B. & Kanneganti, T. D. Advances in Understanding Activation and Function of the NLRC4 Inflammasome. International journal of molecular sciences 22, doi:10.3390/ijms22031048 (2021).

10 Zhang, J. et al. NLRP6 expressed in astrocytes aggravates neurons injury after OGD/R through activating the inflammasome and inducing pyroptosis. International immunopharmacology 80, 106183, doi:10.1016/j.intimp.2019.106183 (2020).

11 Shen, C. et al. Molecular mechanism for NLRP6 inflammasome assembly and activation. Proceedings of the National Academy of Sciences of the United States of America 116, 2052-2057, doi:10.1073/pnas.1817221116 (2019).

12 Kumari, P., Russo, A. J., Shivcharan, S. & Rathinam, V. A. AIM2 in health and disease: Inflammasome and beyond. Immunological reviews 297, 83-95, doi:10.1111/imr.12903 (2020).

13 Derang¨¨re, V. et al. Liver X receptor ¦Â activation induces pyroptosis of human and murine colon cancer cells. Cell death and differentiation 21, 1914-1924, doi:10.1038/cdd.2014.117 (2014).

14 Chen, C. et al. DAC can restore expression of NALP1 to suppress tumor growth in colon cancer. Cell death & disease 6, e1602, doi:10.1038/cddis.2014.532 (2015).

15 Kovarova, M. et al. NLRP1-dependent pyroptosis leads to acute lung injury and morbidity in mice. Journal of immunology (Baltimore, Md. : 1950) 189, 2006-2016, doi:10.4049/jimmunol.1201065 (2012).

16 Guo, X. et al. Benzene metabolites trigger pyroptosis and contribute to haematotoxicity via TET2 directly regulating the Aim2/Casp1 pathway. EBioMedicine 47, 578-589, doi:10.1016/j.ebiom.2019.08.056 (2019).

17 Xia, T. et al. PRMT5 regulates cell pyroptosis by silencing CASP1 in multiple myeloma. Cell death & disease 12, 851, doi:10.1038/s41419-021-04125-5 (2021).

18 Wang, Y. et al. Chemotherapy drugs induce pyroptosis through caspase-3 cleavage of a gasdermin. Nature 547, 99-103, doi:10.1038/nature22393 (2017).

19 Li, Y. et al. GSDME-mediated pyroptosis promotes inflammation and fibrosis in obstructive nephropathy. Cell death and differentiation 28, 2333-2350, doi:10.1038/s41418-021-00755-6 (2021).

20 Jiang, M., Qi, L., Li, L. & Li, Y. The caspase-3/GSDME signal pathway as a switch between apoptosis and pyroptosis in cancer. Cell death discovery 6, 112, doi:10.1038/s41420-020-00349-0 (2020).

21 Khanova, E. et al. Pyroptosis by caspase11/4-gasdermin-D pathway in alcoholic hepatitis in mice and patients. Hepatology (Baltimore, Md.) 67, 1737-1753, doi:10.1002/hep.29645 (2018).

22 Pillon, N. J. et al. Saturated fatty acids activate caspase-4/5 in human monocytes, triggering IL-1¦Â and IL-18 release. American journal of physiology. Endocrinology and metabolism 311, E825-e835, doi:10.1152/ajpendo.00296.2016 (2016).

23 Yang, D., He, Y., Mu?oz-Planillo, R., Liu, Q. & N¨²?ez, G. Caspase-11 Requires the Pannexin-1 Channel and the Purinergic P2X7 Pore to Mediate Pyroptosis and Endotoxic Shock. Immunity 43, 923-932, doi:10.1016/j.immuni.2015.10.009 (2015).

24 Fang, Y. et al. Pyroptosis: A new frontier in cancer. Biomedicine & pharmacotherapy = Biomedecine & pharmacotherapie 121, 109595, doi:10.1016/j.biopha.2019.109595 (2020).

25 He, W. T. et al. Gasdermin D is an executor of pyroptosis and required for interleukin-1¦Â secretion. Cell research 25, 1285-1298, doi:10.1038/cr.2015.139 (2015).

26 Li, L., Li, Y. & Bai, Y. Role of GSDMB in Pyroptosis and Cancer. Cancer management and research 12, 3033-3043, doi:10.2147/cmar.S246948 (2020).

27 Zhou, Z. et al. Granzyme A from cytotoxic lymphocytes cleaves GSDMB to trigger pyroptosis in target cells. Science (New York, N.Y.) 368, doi:10.1126/science.aaz7548 (2020).

28 Hou, J. et al. PD-L1-mediated gasdermin C expression switches apoptosis to pyroptosis in cancer cells and facilitates tumour necrosis. Nature cell biology 22, 1264-1275, doi:10.1038/s41556-020-0575-z (2020).

29 Zhang, J. Y. et al. The metabolite ¦Á-KG induces GSDMC-dependent pyroptosis through death receptor 6-activated caspase-8. Cell research 31, 980-997, doi:10.1038/s41422-021-00506-9 (2021).

30 Shi, H. et al. GSDMD-Mediated Cardiomyocyte Pyroptosis Promotes Myocardial I/R Injury. Circulation research 129, 383-396, doi:10.1161/circresaha.120.318629 (2021).

31 Zhang, Z., Zhang, Y. & Lieberman, J. Lighting a Fire: Can We Harness Pyroptosis to Ignite Antitumor Immunity? Cancer immunology research 9, 2-7, doi:10.1158/2326-6066.Cir-20-0525 (2021).
